# Clustering Rfam 10.1: Clans, Families, and Classes

**DOI:** 10.3390/genes3030378

**Published:** 2012-07-05

**Authors:** Felipe A. Lessa, Tainá Raiol, Marcelo M. Brigido, Daniele S. B. Martins Neto, Maria Emília M. T. Walter, Peter F. Stadler

**Affiliations:** 1 Department of Computer Science, Institute of Exact Sciences, University of Brasília, Brasília 70910-900, Brazil; E-Mail: felipe.lessa@gmail.com; 2 Department of Cellular Biology, Institute of Biology, University of Brasília, Brasília 70910-900, Brazil; E-Mails: tainaraiol@gmail.com (T.R.); brigido@unb.br (M.M.B.); 3 Department of Mathematics, University of Brasília, Brasília 70910-900, Brazil; E-Mail: daniele@mat.unb.br; 4 Bioinformatics Group, Department of Computer Science, and Interdisciplinary Center for Bioinformatics, University of Leipzig, Härtelstraße 16-18, D-04107 Leipzig, Germany; E-Mail: studla@bioinf.uni-leipzig.de; 5 Max Planck Institute for Mathematics in the Sciences, Inselstraße 22, Leipzig D-04103, Germany; 6 Fraunhofer Institut f¨ur Zelltherapie und Immunologie-IZI Perlickstraße 1, D-04103 Leipzig, Germany; 7 Institute for Theoretical Chemistry , University of Vienna, W¨ahringerstraße 17, Wien A-1090, Austria; 8 Center for non-coding RNA in Technology and Health, University of Copenhagen, Grønnegårdsvej 3, DK-1870 Frederiksberg C, Denmark; 9 Santa Fe Institute, 1399 Hyde Park Rd., Santa Fe, NM 87501, USA

**Keywords:** Rfam, non-coding RNA, secondary structure, clans, clusters

## Abstract

The Rfam database contains information about non-coding RNAs emphasizing their secondary structures and organizing them into families of homologous RNA genes or functional RNA elements. Recently, a higher order organization of Rfam in terms of the so-called clans was proposed along with its “decimal release”. In this proposition, some of the families have been assigned to clans based on experimental and computational data in order to find related families. In the present work we investigate an alternative classification for the RNA families based on tree edit distance. The resulting clustering recovers some of the Rfam clans. The majority of clans, however, are not recovered by the structural clustering. Instead, they get dispersed into larger clusters, which correspond roughly to Genes 2012, 3 379 well-described RNA classes such as snoRNAs, miRNAs, and CRISPRs. In conclusion, a structure-based clustering can contribute to the elucidation of the relationships among the Rfam families beyond the realm of clans and classes.

## 1. Introduction

The Rfam database systematically collects sequences, alignments, consensus secondary structures, covariance models (CMs) and the corresponding annotation for RNAs with evolutionarily conserved secondary structures [1,2]. The database is constructed using a small manually curated “seed alignment” for each RNA family which is then expanded by a large-scale search for homologs in nucleotide databases. Rfam-families consist of homologous sequences that can be reasonably well aligned and that share some common function.

Rfam release 10.0 [3] introduced the concept of a *clan* as a means for describing explicit relationships between Rfam families for which homology is recognizable but sequence similarities are too faint for good alignments (such as archaeal RNase P, nuclear RNase P, and the two bacterial RNase P types a and b) or which are classified into different Rfam families because of diverse functions (for example RNAse MRP RNA and the four RNAse P RNA families). Rfam clans thus correspond to the definition of RNA families as used e.g., in [4].

At an even higher level of aggregation, *RNA classes* comprise families that share characteristic sequence and/or structure features, without necessarily being evolutionarily related. The best known examples of RNA classes are animal microRNAs, both classes of small nucleolar RNAs (the box C/D snoRNAs and the box H/ACA snoRNAs) and transfer RNAs. At the level of RNA classes, it is not required for class members to be related by common descent.

There is strong support for the hypothesis that all tRNAs are homologs deriving from a single clover-leaf-structured ancestor [5,6]. Additional RNA families such as mascRNA [7], menRNAs [8], or BC1 [9] are also descendants of tRNAs and hence belong to the tRNA clan. MicroRNA families, on the other hand, frequently arise *de novo* [10,11,12]. In fact, presence/absence patterns of microRNA families have turned out to be a valuable and nearly homoplasy-free phylogenetic marker [13]. It has been argued that novel microRNA families can easily arise in transcribed regions, considering that stem-loop structures resembling microRNA precursors frequently occur in random RNA sequences. This mechanism is most easily seen in the expansion of microRNA clusters by hairpins that are unrelated to more ancient cluster’s components [14]. Topics of innovation and expansion of microRNA families are reviewed e.g., in [15,16]. In regard to snoRNAs it is not clear to what extent families are ancestrally related. While distant homologies can be established in some cases, e.g., U87 and U88 [17], there is also some evidence for the lineage-specific innovation of snoRNA families, e.g., in birds [18, Figure S8].

Clustering of RNAs based on their sequences and/or structural characteristics is probably the simplest approach for identifying families, clans, or classes, see e.g., [19,20,21]. Here we are only concerned with higher levels of aggregation beyond the level of Rfam families; thus we focus on structural similarity. A wide variety of different algorithmic schemes have been proposed to quantify (dis)similarities among known secondary structures. Since secondary structures have canonical representations in the form of ordered trees, tree alignments (RNAforester [22]) and tree editing [23,24] are the most natural and elegant means of comparison. For the specific purpose of clustering, however, it is a fundamental shortcoming of alignments that the cost functions violate the triangle inequality and hence do not form a metric on the set of labeled ordered trees [22]. Hence we employ here a tree-edit distance.

The use of direct structure comparison becomes quite limited in practical applications, because secondary structures of individual sequences are unknown in most cases. Computational prediction of secondary structures for individual sequences, on the other hand, is not sufficiently accurate. This limitation may be overcome, or at least alleviated, however, by using comparative information, see e.g., [25]. Successful applications of RNA clustering [19,21] typically use combined sequence and structure alignments based on the Sankoff algorithm [26] to combine thermodynamic rules with conservation information. For each Rfam family, a high-quality manual sequence alignment together with a matching consensus secondary structure model is available. These consensus structures can be readily used for structure-based clustering.

In this contribution we explore to what extent the manually annotated clans of related Rfam families are detectable by unsupervised clustering, whether RNA classes such as microRNA and the two snoRNA classes are recognizable, and whether there are good candidates for clans or classes of Rfam families that have remained so far undetected.

## 2. Results and Discussion

### 2.1. Clusters and RNA Classes

**Figure 1:**
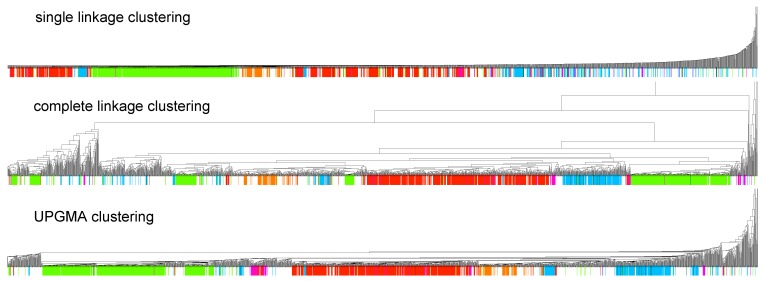
Dendrograms of the consensus structures of all Rfam 10.1 families computed with three different hierarchical clustering methods. Large important classes of ncRNAs are highlighted. Reddish colors denote three classes of microRNAs animal (scarlet), plant (fuchsia), and viral (brown). Box C/D snoRNAs are represented by bright green, while light blue indicates box H/ACA snoRNAs. Prokaryotic CRISPR families are shown in orange.

Starting from the matrix of tree edit distances between the 1973 families collected in Rfam 10.1 we computed hierarchical clusterings using UPGMA [27], single linkage [28], and complete linkage [29] methods. Results for each computation are represented as ultrametric trees (Figure 1). High-resolution versions of all diagrams are available at http://www.biomol.unb.br/rfam/.

Firstly the three hierarchies were compared to each other. By doing this, it was observed that the single linkage hierarchy is clearly different from the other two, considering that it has a caterpillar-like shape with only a few discernible clusters. Figure 1 shows a strong tendency of the microRNA, snoRNA and, to a lesser extent, the CRISPR families to cluster together. All three clustering methods also show a pronounced, although not total, separation among animal, plant, and viral microRNAs. In contrast to viral microRNAs, differences between plant and animal microRNAs have been well recorded in the literature, e.g., reviewed in [30]. Most viral microRNA families in Rfam are from Herpesviridae (16 of 20 viral microRNA families), which clustered with animal microRNA families, as expected. Although viral microRNAs are wide-spread [31,32], they are often poorly conserved and hence have not been included as an Rfam family. 

### 2.2. Clusters and Rfam Clans

**Figure 2:**
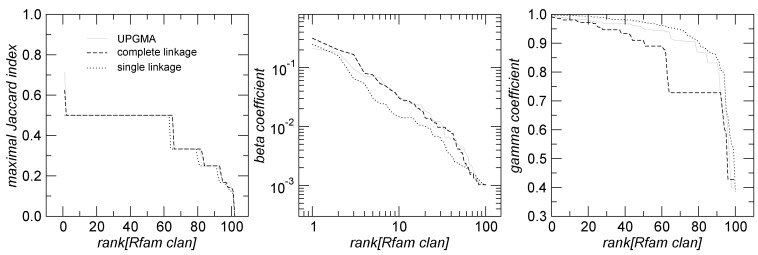
Distribution of *α* (maximal Jaccard index), *β*, and *γ* for all of the 102 Rfam clans. These data show that most clans do not appear tightly clustered w.r.t. any of the three methods. The clans shown in the x-axis, together with *α*, *β*, and *γ* are listed in the supplementary material.

Rfam 10.1 defines 102 clans, the majority of which comprises only two Rfam families. Figure 2 shows that they have not been well recovered by the clustering of the consensus structure. In fact, the coefficient *β* which measures a clan’s tightness within the dendrogram shows a power-law like behavior, indicating that only a few clans are tightly clustered while most clans spread out over large areas of the dendrogram.

This is not entirely unexpected for several distinct reasons. Firstly, many clans consist of structurally related microRNA families. As all animal microRNA precursors share a very similar stem-loop structure, similarities inside a clan are not stronger than those shared with other microRNA families. These clans are thus dispersed throughout the microRNA clusters in Figure 1, leading to small values of α and β. Secondly, this intra-clan similarity clusters most of the animal microRNAs in a single tree branch. In contrast, some other clans combine functionally related families that are clearly structurally distant in their supposed homologous structure: the tRNA clan (CL0001) for instance contains four families of bacterial tmRNAs along with the tRNA and the tRNA-Sec families.

Compared to a tRNA, a tmRNA has a big loop insertion that reflects its role as an mRNA-like repair template for ribosomal protein synthesis. Thus tmRNAs and tRNAs are separated by very large tree-edit distances. The four tmRNA families (RF00023, RF01849, RF01850, RF01851) also feature major differences. The most common one-piece type and distinct families of the two-piece tmRNAs can be respectively found in *α*-proteobacteria, *β*-proteobacteria, and cyanobacteria. In addition, some tmRNAs with a permuted organization have also been described [33,34].

**Figure 3:**
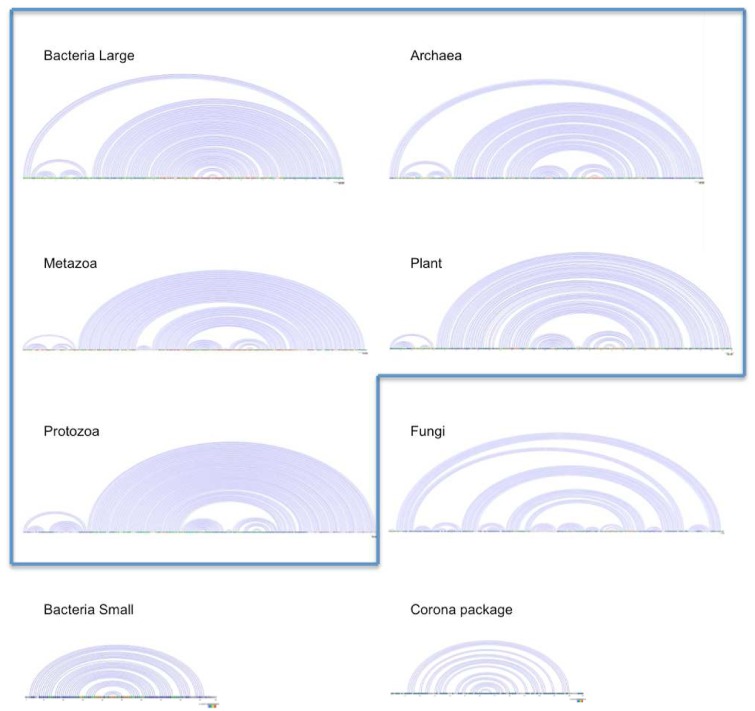
Linear representations of the secondary structures of the SRP clan members. The seven depicted SRP clan members display a conserved stem loop structure. The five of them that appear as a cluster in the UPGMA tree are delimited by a blue boundary. Fungal SRP family contains extra loops, while the Small Bacterial SRP families contain only a conserved stem loop domain, thus they have been both excluded from the cluster. An UPGMA neighbor of the Small Bacterial SRP family, an unrelated virus derived RFAM family (Corona package), is shown for comparison.

Despite the usually poor representation of Rfam clans in the structure-based clustering, there are a few clans that at least partially agree with the clustering data. The SRP clan (CL00003) is the one that was most coherently recovered as measured by the maximal Jaccard index α ≈ 0.71. It consists of 5 phylogenetically defined subgroups of signal recognition particle RNAs, whose homology is well established [35]: Metazoa SRP (RF00017), Bacteria large SRP (RF01854), Plant SRP (RF01855), Archaea SRP (RF01857) and Protozoa SRP (RF01856) (Figure 3). In addition to these families, which cluster together in our analysis, the SRP clan also contains Bacteria small SRP (RF00169) and Fungi SRP (RF01502), which were located far apart from the clustered SRP families in the UPGMA tree. The bacterial small SRP RNA (4.5S RNA) family (RF00169) is fully functional in mycoplasma and gram-negative bacteria, and harbors the conserved helices 5 and 8, which can be found in all kingdoms [35]. The reduced size of such RNAs prevented them from clustering with other SRP families. In our analysis, they clustered with RF00182, an unrelated viral element without any obvious functional or phylogenetic association. The fungal SRP RNA components (RF01502), on the other hand, possessed a highly conserved structure when compared to the other clan member. It contains several extra helices, however, that explain the large values of the tree distance in comparison to the other clan members. Thus, it appeared separated in a distinct tree branch. This case exemplifies that clustering with global distance functions alone cannot cope with those cases where there are dramatic structural differences between homologous RNAs.

Therefore, tree edit distance does not necessarily respect phylogenetic relationships. Nevertheless, homologous structures are frequently located in close proximity in the tree. Ribonuclease P, an ubiquitous ribozyme required for tRNA processing [36,37], for instance, is represented by four Rfam families in the clan (CL0002). Although they are located within the same subtree, they cluster with functionally and evolutionarily unrelated Rfam families such as fungi_U3 (RF01846), U1_yeast (RF00488), and the internal ribosomal entry sites of hepatitis A virus, IRES_HepA (RF00228). Interestingly, the alpha_tmRNA is also found in the RNAse P sub-tree, very close to RNAseP_nuc (RF00009) and RUF21 (RF01825), an yeast ncRNA with unknown functions. On the other hand, the prototypic tmRNA (RF00023) clusters together with the RNAse_MRP (RF00030), a distant homolog of RNAse P [38,39]. Therefore, one can speculate that RNAse P and tmRNA might share a common evolutionary history.

It is worth to mention that functional similarity, on the other hand, does necessarily imply structural similarity. The IRES structures, frequently found in RNA virus and several cellular genes [40], are fine examples, although there are neither common designs, nor common signatures, or even common origins. Therefore, it is not surprising that they are found dispersed all over the cluster tree, gathering diverse functionally unrelated families.

The large plateau at 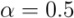 in Figure 2 mostly consists of size two clans, which are not recovered as cherries in the clustering tree. The CRISPR-2 clan is among the few larger clans that cluster together with most of its families. Using the dispersion coefficient *β* as a measure, clans CRISPR-2 and CRISPR-1 are well clustered. In addition a few microRNA and snoRNA clans with only two members received relatively large *β* values. A visual inspection of the UPGMA tree (Figure 1) reveals that differences in structural complexity can be directly inferred from the tree: larger and more complex RNAs, in particular rRNAs, appear isolated in this tree’s leftmost. In the other extreme, simple structural elements such as microRNA’s precursor hairpins and snoRNAs’ simple structures were clearly clustered together.

### 2.3. Significant Structure-Based Clusters

It is natural to ask whether the hierarchical clustering trees can be used to identify significant structure-based clusters, and whether the families with strong similarities share biological functions. The Rfam clans have been constructed manually and consist of groups of families sharing common ancestors too divergent to be aligned, or also presenting good alignments but distinct functions so that they could not be included in the same Rfam family [3]. Some of these clans clearly share a common evolutionary ancestry, considering that similarities in their biological functions were experimentally verified. Hierarchical clustering, on the other hand, suggests how Rfam families that are not contained in clans could be related to each other and how clans may be organized at even higher levels of aggregation. It thus provides a more inclusive annotation.

**Figure 4:**
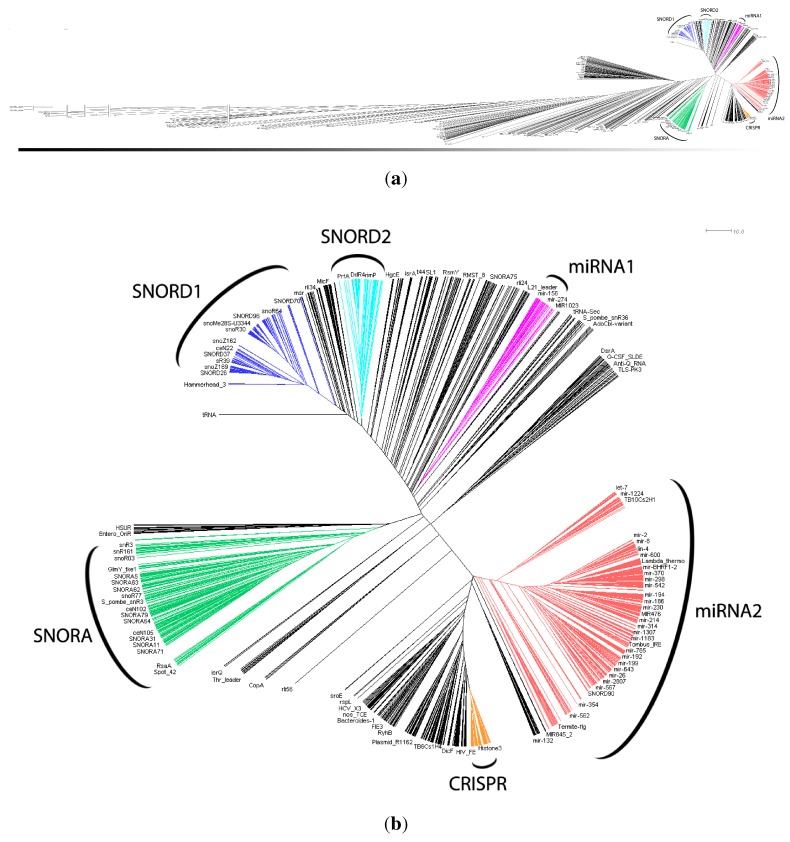
Circular view of the UPGMA structural distance-based tree. (**a**) Circular view of the complete UPGMA dendrogram. One can see below the tree a gradient indicating more complex structures on the left and simpler ones on the right. Vertical bars represent shortening of branch length; (**b**) Closer view of clustered snoRNAs (SNORD1 (dark blue), SNORD 2 (light blue) and SNORA (green)), miRNAs (miRNA1 (pink) and miRNA2 (red)) and CRISPR (orange).

Generally speaking, clan families were not tightly clustered together by our structure-based distance trees. In particular, larger, more complex secondary structures appeared widely separated from smaller, simpler ones (Figure 4(a)). Figure 4(b) shows in more detail the right hand side of Figure 4(a). As expected, C/D box snoRNAs (SNORD), H/ACA box snoRNAs (SNORA), miRNAs, and CRISPRs were clustered tightly together. Our data suggest then six clusters: SNORD1, SNORD2, SNORA, miRNA1, miRNA2 and CRISPR. The families included in these clusters are listed in Table 1. Such clusters possess very simple and clear secondary structure relationships and aggregate a large fraction of the families in the Rfam database.

## 3. Experimental Section

Stockholm formatted alignments were retrieved as well as some metadata (such as accession numbers and short descriptions) for each of the 1,973 families of the Rfam database version 10.1 [3]. Then, the consensus structure was extracted from each alignment. Both Rfam and Vienna RNA Package [41] use string representations for secondary structures in which each base pair is denoted by a matching pair of parentheses and each unpaired bases by dots. This string has a natural interpretation as an ordered forest: dots denote leaf nodes and pairs of parentheses, interior nodes.

In this study, a full tree editing distance was used, in which every unpaired base and base pair were represented as nodes in the tree representation of the secondary structure. Canonical edit operations for forests were done by insertions and deletions of nodes. Upon node’s deletion, its children became parent-node’s children. Interior nodes represented base pairs and hence two nucleotides, while leaf nodes referred to single nucleotides, therefore in/del costs were set to 2 for interior nodes and 1 for leaves. Edit distance 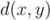 of two ordered forests 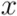 and 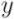 can in general be computed efficiently by means of a dynamic programming algorithm [42]. Here we used an implementation available in the RNAdistance program, a component of the Vienna RNA Package (version 1.8.4). One disadvantage of using this distance measure is that two secondary structures of very distinct lengths always become very distant, even if they possess similar motifs. In addition, one of the most time consuming steps was running RNAdistance, since almost 2 million structure comparisons had to be completed. Nevertheless, this procedure took only a few hours.

**Table 1:** Clusters of snoRNAs, miRNAs, and CRISPRs.

Cluster	Number of Rfam families included	Percentage of Rfam families of the expected ncRNA	Clans (name and identiﬁcation) with all families included in *Cluster*
SNORD1	334	94.9%	SNORD52 (CL00063), U54 (CL00008), SNORD26 (CL00050),
			SNORD44 (CL00060), SNORD58 (CL00064), SNORD101 (CL00074),
			SNORD105 (CL00075), SNORND104 (CL00077)
			SNORD61 (CL00067), SNORD39 (CL00057), SNORD18 (CL00047),
			SNORD34 (CL00055), SNORD96 (CL00072), SNORD110 (CL00076),
			SNORD30 (CL00052), SNORD19 (CL00048), SNORD100 (CL00073)
SNORD2	86	81.4%	SNORD15 (CL00045)
SNORA	158	81.0%	SNORA7 (CL00025), SNORA28 (CL00033), SNORA44 (CL00036),
			SNORA17 (CL00029), SNORA35 (CL00034), SNORA5 (CL00024),
			SCARNA4 (CL00019)
miRNA1	45	86.6%	MIR171 (CL00099)
miRNA2	472	85.6%	mir-34 (CL00087), mir-216 (CL00094), mir-279 (CL00095),
			mir-36 (CL00088), mir-81 (CL00091), mir-182 (CL00093),
			mir-3 (CL00084), mir-50 (CL00089), mir-BART (CL00097),
			mir-137 (CL00092), mir-73 (CL00090)
CRISPR	100	59.0%	CRISPR-1 (CL00014), CRISPR-2 (CL00015)

Various agglomerative clustering methods differ only in their definition of the distance measure 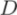 between clusters. In each step, the two closest clusters, 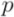 and 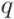, are united to a single cluster 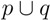. The distance of 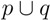. to all clusters 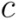 is then obtained recursively starting from 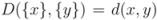 for clusters consisting of individual points. The form of the recursion determines the particular clustering method [43]. For UPGMA 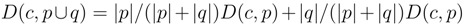, for single linkage 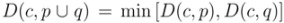, and for complete linkage 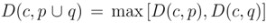. The resulting hierarchy of clusters is conveniently represented as a dendrogram 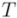, in which leafs are the individual points. Each cluster 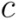 is uniquely identified by a node 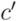 as the set of leafs of subtree rooted at 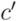. Dendrograms are drawn with a custom-made tool that allows to highlight sets of leaves using regular expressions that match against the extracted Rfam metadata.

Distance-based clusters are compared to Rfam clans and some other groupings, such as microRNAs or snoRNAs, using three quantitative measures. Denote 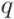 by an externally defined group, and let 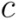 be a cluster of 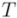. Then we define

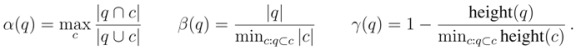
(1)
where 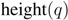 is the height of a given subtree 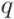 as calculated by the cluster distance 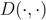, *i.e*., zero for a leaf 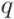, or 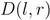 for a subtree 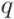 having children 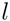 and 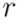. The maximal Jaccard index 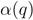 compromises between coverage and contamination. 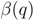 measures how dispersed 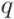 is in 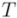 by computing the fraction of members of 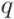 which compose the smallest cluster 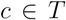 that entirely contains 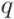. This measure is quite sensitive to individual outliers. On the other hand, 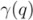, which is also a measure of dispersion, takes the dendrogram cluster heights avoiding to assign bad scores to groups of very similar families. If the pre-defined group 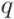 appears as a cluster in 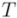, 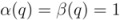.

The proposed clusters were constructed under careful examination of the most representative Rfam families of a particular ncRNA in the UPGMA dendrogram, *i.e*., a cluster was formed containing the largest number of relatively close Rfam families in the tree. The parsing of the Rfam, the hierarchical clustering, and the drawing of the resulting dendrograms were implemented in Haskell, the source code of which is available at http://hackage.haskell.org/package/ under the open source 3-clause BSD license. As mentioned before, the most time-consuming step was the computation of the distance matrix with RNAdistance, while all the other computations took only a few minutes on a notebook.

## 4. Conclusions

We have developed a tool that calculates and draws automatically dendrograms showing the relationship among all the Rfam’s ncRNA families. Such dendrograms have demonstrated to be able to confirm already expected relationships, such as snoRNAs and microRNAs, and also to be able to expose unknown ones, such as those discussed in Section 2.2.

The computational clustering reported here includes all of the 1,973 Rfam families, compared to only 306 families that were manually annotated as members of Rfam clans. Our analysis suggests that the automatic and manual methods should be combined to comprehensively reveal the structural and evolutionary relationships of the entire content of the Rfam database.

As future work, the dendrograms could be further analyzed, considering that it is suspected that there may be more information to be extracted than what we have already covered with this work. We are interested in studying putative ncRNAs’ classes that can be defined based on the information we obtained from the dendrograms. For instance, to measure the clusters’ consistency derived from those trees, it would be useful to compute the probability of finding the correct cluster for a given sequence. This could be used to know if a particular clustering could be used to predict a snoRNA or a miRNA class based on the secondary structure. It would also be interesting to see if there is a biological basis for the appearance of two clusters of box C/D snoRNAs and microRNAs, respectively, in Figure 4(b). Finally, there is also room for testing different distance measures, including ones that are less affected by length differences or measures that explicitly take into account conserved sequence elements.

## Supplementary Files

Supplementary File 1 PDF-Document (PDF, 64 KB)

Supplementary File 2 PDF-Document (PDF, 58 KB)

Supplementary File 3 PDF-Document (PDF, 44 KB)
