# Supplementary material for: Clustering Rfam 10.1: Clans, Families, and Classes
Source: Genes (Basel). 2012 Jul 5;3(3):378–90. doi: 10.3390/genes3030378 (PMC3899987; doi:10.3390/genes3030378)
Supplement: Supplementary File 1 — PDF-Document (PDF, 64 KB) [file genes-03-00378-s001.pdf]

List of clusters

Cluster SNORD1: 334 families, of which 94.9% are SNORDs, contains 74.4% of all SNORDs.

Families: Hammerhead_3, SNORD56, CAESAR, SNORD26, SNORD41, SNORD93, SNORD66, snoR10, sn2429, ceN89, snoR60, SNORD107, sn3071, SNORD42, SNORD82, SNORND104, snoU49, snoMe28S-Cm788, snoU43C, SNORD50, sn2317, plasmodium_snoR24, snoR28, plasmodium_snoR30, plasmodium_snoR17, snoZ175, plasmodium_snoR28, SNORD31, SNORD102, SNORD109A, snoZ17, SNORD99, SNORD112, SNORD52, SNORD75, ceN53, ceN108, sn2841, SNORD108, ceN109, ceN111, ceN70, ceN30, ceN44, snoU54, ceN61, SNORD51, SNORD115, snoJ33, snoZ155, HIV_GSL3, SNORD72, SNORD105, snoR66, SNORD95, snoR79, sn3060, snoZ169, SNORD123, snoZ267, SNORD126, snoZ40, SNORD98, sn1185, snoR25, SNORD69, sn668, snoPyro_CD, ceN114, ceN65, SNORD44, ceN63, SNORD48, sR4, ceN54, sR24, sR58, sR45, sR60, sR20, sR53, sR22, sR40, sR32, sR47, sR13, sR38, sR36, sR48, sR39, sR16, sR14, snoU82P, sR52, sR7, sR23, sR2, sR33, sR44, sR28, sR51, sR35, sR43, sR30, sR18, sR17, sR49, sR41, sR10, sR8, sR34, SNORD37, SNORD78, DdR17, sR9, snoR101, sR42, sR15, sR11, v-snoRNA-1, DdR14, sR19, sR1, sR5, sR21, sR12, sR55, DdR1, SAM_V, snoR43, SNORD38, snoR4a, ceN22, ceN27, PRINS, SNORD83, snoR72, SCARNA18, snoMe28S-G3255, snoCD11, Lnt, SAM_alpha, PreQ1, snoZ162, Bacillus-plasmid, snoR30, SNORD36, snoR117, snoZ6, sn2903, SNORD27, snoR12, snoMe28S-Gm3113, SNORD29, snoZ165, snoU83, snoR09, SNORD61, snoU83D, snoR07, snoMe28S-Am982, snoU83C, snoR121, snoR24, SNORD57, SNORD11, snoR20a, snoU31b, snoU30, snR50, SNORD21, snoMe28S-Gm1083, SNORD35, snoR77Y, snoZ196, snoR27, snoZ173, snoR26, snoR31_Z110_Z27, snoR160, U54, snoZ163, SNORD111, snoR11, snoU83B, sn2991, snR39B, snoR16, snoR8a, SNORD25, SNORD101, sn1502, SNORD73, SNORD39, SNORD28, SNORD62, snoMe28S-Cm3227, snoR29, ceN28, snoZ168, SNORD20, SNORD45, SNORD47, snoR22, SNORD110, sn2343, SNORD74, snoZ7, ceN106, SNORD59, snoR23, snoMe28S-Cm2645, snoMe18S-Gm1358, snoR01, snoMe28S-U3344, snoZ256, snoR18, snoZ223, snoZ122, SNORD79, SNORD87, SNORD103, SNORD100, SNORD81, snoR21, SNORD127, SNORD34, snoZ185, snoZ182, SNORD11B, SNORD12, SNORD30, DdR11, SNORD65, sR3, DdR12, snoZ105, snoU18, DdR2, SNORD49, SNORD5, Afu_199, snoR4, DdR13, DdR16, snoU105B, snoR35, DdR10, DdR5, plasmodium_snoR20, SNORD19B, SNORD19, snoTBR7, SNORD96, snoR53Y, snoZ103, ceN69, snoMBII-202, SNORD113, SNORD43, snoR114, snoR20, snoR14, snoR13, snoR44_J54, snoR32_R81, snoMe28S-Am2634, snoR41, SNORD46, SNORD24, SNORD18, snoU61, snosnR57, snosnR71, snoU36a, snoMe18S-Um1356, snoR128, snR62, SNORD58, SNORD63, snoR116, snoZ159, snoR72Y, snoR31, snoR19, snoTBR5, snR78, snoZ30a, SNORD53_SNORD92, snosnR55, snR58, Afu_335, DdR7, Afu_298, Afu_455, MIR478, snR52, Afu_300, snoU43, snR77, snoR64a, DdR6, Afu_294, DdR8, snoZ161_228, snoZ43, SNORD33, snoR38, SNORD88, snoZ266, snoR71, SNORD60, snoR64, snoZ101, snoZ102_R77, SNORD91, SNORD124, SNORD125, snoZ199, snoZ278, snoU6-53, SNORD116, snoZ30, snoZ118, HOTAIR_3, SNORD70, SNORD121A, c-di-GMP-II, rli27, HSR-omega_2, RatA, msr, snoZ157, snoZ119.

Cluster SNORD2: 86 families, of which 81.4% are SNORDs, contains 16.4% of all SNORDs.

Families: SNORD14, PrfA, S15, Alpha_RBS, snoZ221_snoR21b, snoR9_plant, plasmodium_snoR14, ceN113, snoZ5, sn2417, snR13, plasmodium_snoR16, snosnR60_Z15, snoZ107_R87, snoZ206, snoZ152, snoZ188, snR76, snosnR64, snR56, Afu_304, DdR15, snoU13, Afu_191, snR79, snR51, snoU40, snR41, ceN103, snoR113, snoR130, ceN40, snosnR48, ceN33, snosnR69, snoR1, snoR17, sn2524, snoR126, snosnR66, snoR69Y, SNORD23, snosnR54, Afu_514, Afu_513, DdR4, snR73, snosnR61, snR40, Afu_264, snR47, Afu_190, snR65, snoR118, snoMe28S-Am2589, snR39, snR87, bxd_4, MALAT1, snoZ247, snoJ26, bxd_3, PhotoRC-II, RMST_4, Hammerhead_1, rimP, SNORD15, Afu_198, ceN47, SNORD22, SNORD94, snoR127, plasmodium_snoR21, snoU6-47, snR67, snR75, snR68, snoZ13_snr52, plasmodium_snoR26, snoU6-77, Afu_254, CDKN2B-AS, SAH_riboswitch, U6atac, SNORA29, SMK_box_riboswitch.

Cluster SNORA: 158 families, of which 81.0% are SNORAs, contains 57.1% of all SNORAs.

Families: Spot_42, OxyS, CC1840, CC3552, VrrA, RsaE, greA, HLE, snoR111, frnS, RsaA, rli49, SNORA71, SNORA40, SNORA4, SNORA68, SCARNA3, ceN92, SNORA25, snoF1_F2, S_pombe_snR93, SNORA42, SNORA49, S_pombe_snR92, SCARNA20, ceN49, ceN68, ceN84, SNORA11, SCARNA8, SNORA24, ceN88, ceN125, SNORA65, ceN41, SNORA36, SNORA51, SNORA56, ceN43, ceN86, SNORA31, SNORA3, ceN100, ceN80, ceN93, ceN81, ceN58, SNORA20, ceN38, ceN126, ceN48, ceN67, ceN101, ceN39, ceN105, SNORA30, ceN45, SNORA76, ceN42, ceN51, DdR18, SCARNA23, 23S-methyl, rli61, Pxr, SNORA64, SNORA7, SNORA48, SNORA84, ceN82, SNORA69, SNORA55, SNORA15, SCARNA4, SNORA79, SNORA58, SNORA35, SNORA77, ceN110, SCARNA15, SNORA47, snopsi28S-3327, snoR134, snopsi28S-1192, snoR74, snoR137, ceN102, snopsi28S-3316, SNORA67, SCARNA21, SNORA13, SNORA54, SNORA14, SNORA46, S_pombe_snR3, RUF2, SNORA28, snoR80, SNORA32, ACA64, ceN46, snoR104, S_pombe_snR33, snoR77, SCARNA11, SCARNA14, SNORA8, SNORA17, SNORA62, SNORA50, SNORA43, snoR639, SNORA1, SNORA19, SNORA33, snoU109, ceN104, snR85, SNORA63, SNORA21, SNORA41, ceN36-1, S_pombe_snR90, snopsi18S-1854, SNORA52, SNORA38, S_pombe_snR5, SNORA72, SNORA9, SNORA5, SNORA66, SNORA44, SNORA70, SNORA57, SNORA61, S_pombe_snR46, GlmY_tke1, plasmodium_snoR27, SNORA22, SNORA18, Termite-leu, snopsi18S-841, snoR03, snR49, SNORA23, snR33, snR80, snR161, S_pombe_snR10, snR9, S_pombe_snR35, snR5, S_pombe_snR42, snR81, snR189, snR3, snopsi28S-2876, NrrF.

Cluster miRNA1: 45 families, of which 53.3% are plant miRNAs, contains 43.6% of all plant miRNAs.

Families: Vault, MIR480, Y_RNA, L21_leader, MIR394, MIR390, MIR398, MIR408, mir-172, MIR171_1, MIR168, MIR812, mir-156, MIR477, MIR162_2, MIR171_2, mir-11, MIR473, MIR828, MIR529, MIR403, mir-160, mir-28, MIR397, mir-399, mir-395, mir-689, K_chan_RES, rli51, MIR475, mir-277, mir-544, mir-3017, mir-286, mir-584, mir-274, MIR474, MIR1122, mir-649, Deinococcus_Y_RNA, MIR169_5, MIR444, MIR2118, MIR530, MIR1023.

Cluster miRNA2: 472 families, of which 85.6% are animal miRNAs, contains 91.6% of all animal miRNAs.

Families: let-7, mir-284, mir-306, mir-324, mir-218, mir-449, mir-995, mir-996, mir-684, mir-24, mir-302, mir-71, mir-67, ceN74-2, rox2, mir-1224, mir-433, RNA-OUT, mir-290, mir-16, mir-33, mir-235, Rota_CRE, HHBV_epsilon, mir-877, mir-133, mir-23, mir-216, mir-146, mir-147, mir-245, potC, TB10Cs2H1, TB11Cs4H3, TB10Cs5H3, TB10Cs5H2, TB9Cs1H2, mir-384, mir-1275, mir-2, mir-103, mir-425, mir-2241, mir-17, mir-122, mir-203, mir-9, mir-221, mir-460, mir-96, mir-208, mir-181, mir-10, mir-30, F6, mir-101, mir-155, mir-308, mir-486, mir-14, mir-8, mir-46, mir-130, mir-3, mir-927, mir-92, mir-231, mir-1, mir-138, mir-19, mir-887, lin-4, mir-242, mir-874, mir-29, mir-790, mir-134, mir-361, mir-135, mir-196, mir-128, mir-499, mir-148, mir-791, mir-872, mir-920, mir-263, mir-275, mir-232, mir-600, mir-500, mir-TAR, mir-378, rox1, HBV, mir-484, mir-62, mir-572, mir-1827, GABA3, SECIS_2, Lambda_thermo, mir-BART1, mir-1829, mir-761, mir-557, mir-BART2, mir-139, Gurken, mir-BHRF1-1, mir-BHRF1-3, g2, mir-383, mir-760, mir-673, mir-1255, mir-BHRF1-2, mir-318, mir-392, mir-458, mir-5, mir-12, mir-BART20, mir-BART3, lsy-6, mir-21, mir-367, mir-675, mir-1473, mir-32, mir-299, mir-1180, mir-374, mir-668, mir-671, mir-503, mir-60, mir-1226, mir-250, mir-879, mir-875, mir-1912, mir-488, mir-281, mir-370, mir-105, mir-451, mir-883, mir-885, mir-455, mir-224, ciona-mir-92, mir-185, mir-574, mir-136, mir-197, mir-506, mir-491, mir-592, mir-61, mir-207, MIR815, mir-938, mir-149, mir-BART7, mir-941, mir-298, mir-422, mir-1287, mir-939, mir-1227, SNORD86, mir-BART15, mir-631, mir-322, mir-604, mir-708, mir-504, mir-200, mir-1280, mir-542, mir-662, mir-289, mir-52, mir-150, mir-304, mir-940, mir-331, mir-583, mir-563, mir-194, mir-541, mir-190, mir-305, mir-BART5, mir-34, mir-317, mir-153, mir-145, MIR845_1, mir-315, mir-787, mir-330, mir-676, mir-551, mir-569, mir-365, mir-489, mir-259, mir-351, mir-186, mir-581, mir-609, mir-42, mir-279, mir-50, mir-244, mir-43, MIR2587, mir-22, mir-423, mir-786, mir-191, MIR1444, mir-326, mir-230, mir-942, mir-934, mir-625, mir-944, mir-3179, mir-605, mir-1265, mir-578, mir-86, mir-253, mir-296, mir-505, mir-316, mir-452, mir-876, mir-359, mir-580, mir-548, MIR439, mir-80, mir-789, mir-90, mir-228, MIR476, mir-586, mir-576, mir-642, mir-577, mir-624, mir-651, mir-932, mir-616, mir-339, mir-335, mir-802, mir-618, mir-357, mir-246, mir-248, mir-251, mir-214, mir-552, mir-599, mir-77, mir-81, mir-75, mir-49, mir-628, mir-BART12, mir-558, mir-621, mir-340, mir-314, mir-1307, mir-597, Actino-pnp, mir-650, mir-154, mir-922, mir-1183, mir-615, mir-648, PYLIS_1, mir-640, mir-936, mir-663, MIR1428, mir-593, Tombus_IRE, mir-1207, mir-1208, mir-25, mir-937, mir-198, mir-7, mir-744, mir-632, mir-288, mir-589, mir-612, mir-765, mir-770, MIR1446, mir-63, mir-607, mir-1237, mir-767, mir-1253, mir-498, mir-192, mir-654, mir-183, mir-193, mir-239, mir-929, mir-345, mir-711, mir-320, mir-633, hvt-mir-H, mir-199, mir-450, mir-144, mir-432, mir-202, mir-556, mir-1388, mir-337, mir-549, mir-74, mir-672, mir-233, mir-434, mir-988, mir-342, mir-350, mir-360, mir-142, mir-85, mir-653, mir-83, mir-137, MIR158, MIR1846, mir-643, mir-2024, mir-BART17, mir-764, mir-987, mir-1249, MIR535, mir-268, mir-665, mir-2778, mir-652, mir-983, mir-573, mir-129, bantam, mir-210, mir-346, mir-328, mir-590, mir-582, mir-26, mir-219, mir-182, mir-338, mir-27, mir-553, mir-2774, mir-1251, mir-375, mir-463, mir-891, mir-126, mir-M7, mir-iab-4, mir-540, mir-471, mir-363, mir-692, mir-204, mir-240, mir-355, mir-73, mir-344, mir-550, mir-2807, mir-223, mir-456, mir-217, mir-287, mir-644, mir-140, mir-55, mir-234, mir-674, mir-1296, mir-254, mir-3180, mir-143, mir-70, mir-127, mir-412, mir-280, mir-639, mir-431, mir-497, mir-567, mir-575, mir-6, mir-44, mir-283, mir-276, mir-278, mir-241, mir-184, mir-282, mir-58, mir-188, mir-36, mir-64, nuoG, mir-981, mir-87, mir-358, SNORD90, mir-1178, mir-2238, mir-448, mir-48, mir-969, mir-354, mir-636, mir-492, Xist_exon4, mir-562, Parecho_CRE, rli31, mir-720, mir-454, Bacteria_small_SRP, Corona_package, Retro_dr1, mir-657, Lacto-usp, mir-626, mir-661, Termite-flg, lactis-plasmid, mir-205, mir-187, mir-598, mir-84, mir-490, mir-353, mir-255, ceN23-1, mir-2518, MIR405, mir-249, mir-999, MIR1027, MIR1222, mraW.

Cluster CRIPSR: 100 families, of which 59.0% are CRISPRs, contains 90.8% of all CRISPRs.

Families: Histone3, CRISPR-DR14, CRISPR-DR42, CRISPR-DR43, CRISPR-DR38, CRISPR-DR55, CRISPR-DR60, HOTAIR_1, cHP, sR6, RF_site1, RF_site3, CRISPR-DR32, CRISPR-DR47, CRISPR-DR62, TLS-PK6, CRISPR-DR7, CRISPR-DR64, CRISPR-DR10, CRISPR-DR5, CRISPR-DR46, CRISPR-DR33, CRISPR-DR36, CRISPR-DR30, CRISPR-DR63, CRISPR-DR18, CRISPR-DR13, CRISPR-DR24, CRISPR-DR52, CRISPR-DR19, CRISPR-DR53, CRISPR-DR59, CRISPR-DR48, RF_site8, CRISPR-DR49, RF_site9, bxd_5, REN-SRE, PK-BYV, CRISPR-DR22, eiav_FSE, fiv_FSE, CRISPR-DR58, TMV_UPD-PK3, PK1-TEV_CVMV, JUMPstart, SBWMV1_UPD-PKe, blv_FSE, BMV3_UPD-PK1, CRISPR-DR45, UPD-PKc, SBWMV2_UPD-PKl, SBWMV1_UPD-PKb, SBRMV1_UPD-PKf, SBWMV2_UPD-PKb, SBRMV1_UPD-PKd, SBWMV2_UPD-PKk, PSLVbeta_UPD-PK2, TMV_UPD-PK2, BMV3_UPD-PK3, TMV_UPD-PK1, Corona_SL-III, CRISPR-DR11, CRISPR-DR9, CRISPR-DR29, CRISPR-DR20, CRISPR-DR16, CRISPR-DR66, CRISPR-DR21, CRISPR-DR57, CRISPR-DR8, SECIS_4, CRISPR-DR2, CRISPR-DR51, CRISPR-DR3, CRISPR-DR39, CRISPR-DR44, CRISPR-DR26, CRISPR-DR37, CRISPR-DR54, UPSK, SBWMV1_UPD-PKh, UPD-PK2, UPD-PKg, UPD-PKib, CRISPR-DR4, HIV-1_SL3, HIV-1_SL4, CRISPR-DR17, CRISPR-DR25, CRISPR-DR28, TCV_Pr, CRISPR-DR35, CRISPR-DR6, CRISPR-DR27, CRISPR-DR23, CRISPR-DR61, CRISPR-DR15, CRISPR-DR56, CRISPR-DR65.
