# Supplementary material for: Clustering Rfam 10.1: Clans, Families, and Classes
Source: Genes (Basel). 2012 Jul 5;3(3):378–90. doi: 10.3390/genes3030378 (PMC3899987; doi:10.3390/genes3030378)
Supplement: Supplementary File 2 — PDF-Document (PDF, 58 KB) [file genes-03-00378-s002.pdf]

Alpha, beta and gamma values for all clans

ClanAC	ClanID	alpha	beta	gamma
CL00003	SRP	0,71428571	0,00356234	0,58360825
CL00015	CRISPR-2	0,5	0,21052632	0,99272328
CL00019	SCARNA4	0,5	0,18181818	0,97074271
CL00048	SNORD19	0,5	0,0952381	0,99365403
CL00024	SNORA5	0,5	0,07142857	0,96057036
CL00089	mir-50	0,5	0,06896552	0,98191669
CL00099	MIR171	0,5	0,04444444	0,95002992
CL00090	mir-73	0,5	0,02898551	0,97720389
CL00097	mir-BART	0,5	0,025	0,98263434
CL00007	U4	0,5	0,02439024	0,91554699
CL00092	mir-137	0,5	0,02409639	0,97694778
CL00006	U2	0,5	0,02352941	0,91341612
CL00073	SNORD100	0,5	0,02325581	0,99249899
CL00052	SNORD30	0,5	0,01851852	0,99158521
CL00029	SNORA17	0,5	0,01612903	0,95853922
CL00084	mir-3	0,5	0,01351351	0,97939706
CL00025	SNORA7	0,5	0,01282051	0,94878695
CL00047	SNORD18	0,5	0,01212121	0,98895276
CL00055	SNORD34	0,5	0,01212121	0,98895276
CL00072	SNORD96	0,5	0,01212121	0,98895276
CL00076	SNORD110	0,5	0,01212121	0,98895276
CL00008	U54	0,5	0,00615385	0,98240714
CL00050	SNORD26	0,5	0,00615385	0,98240714
CL00060	SNORD44	0,5	0,00615385	0,98240714
CL00064	SNORD58	0,5	0,00615385	0,98240714
CL00074	SNORD101	0,5	0,00615385	0,98240714
CL00075	SNORD105	0,5	0,00615385	0,98240714
CL00077	SNORND104	0,5	0,00615385	0,98240714
CL00088	mir-36	0,5	0,00503778	0,97282105
CL00091	mir-81	0,5	0,00503778	0,97282105
CL00087	mir-34	0,5	0,00451467	0,96715421
CL00094	mir-216	0,5	0,00451467	0,96715421
CL00095	mir-279	0,5	0,00451467	0,96715421
CL00010	Hammerhead	0,5	0,00434783	0,96735847
CL00049	SNORD25	0,5	0,00434783	0,96735847
CL00079	snR68	0,5	0,00434783	0,96735847
CL00081	snoU13	0,5	0,00397614	0,95562708
CL00020	SL	0,5	0,00325733	0,94515921
CL00085	mir-15	0,5	0,00243902	0,9617419
CL00016	FinP-traJ	0,5	0,00231214	0,94689386
CL00100	U3	0,5	0,0020377	0,66457625
CL00046	SNORD16	0,5	0,00133511	0,94367721
CL00070	SNORD77	0,5	0,00133511	0,94367721
CL00080	snoR53	0,5	0,00133511	0,94367721
CL00086	mir-28	0,5	0,00133511	0,94367721
CL00018	SCARNA3	0,5	0,00119976	0,93967039
CL00030	SNORA20	0,5	0,00119976	0,93967039
CL00011Glm		0,5	0,00115741	0,90999479
CL00026	SNORA8	0,5	0,00115741	0,90999479
CL00009	U6	0,5	0,00109709	0,90576014
CL00012	SAM	0,5	0,00109709	0,90576014
CL00039	SNORA56	0,5	0,00109709	0,90576014
CL00044	SNORD12	0,5	0,00109709	0,90576014
CL00059	SNORD43	0,5	0,00109709	0,90576014

CL00043	SNORA74	0,5	0,00107411	0,87194524
CL00022	SNORA3	0,5	0,00107009	0,86744392
CL00023	SNORA4	0,5	0,00107009	0,86744392
CL00017	IRES1	0,5	0,00103896	0,83190149
CL00061	SNORD46	0,5	0,00103896	0,83190149
CL00082	snoU85	0,5	0,00103896	0,83190149
CL00098	MIR169	0,5	0,00103896	0,83190149
CL00101	Cobalamin	0,5	0,00103896	0,83190149
CL00041	SNORA64	0,5	0,0010352	0,80766785
CL00078	snR30-U17	0,5	0,00102775	0,71320381
CL00013	7SK	0,5	0,00101885	0,66457625
CL00037	SNORA48	0,5	0,00101729	0,47470574
CL00036	SNORA44	0,33333333	0,02564103	0,94878695
CL00033	SNORA28	0,33333333	0,01923077	0,94878695
CL00067	SNORD61	0,33333333	0,01734104	0,98586023
CL00056	SNORD35	0,33333333	0,00652174	0,96735847
CL00058	SNORD41	0,33333333	0,00652174	0,96735847
CL00065	SNORD59	0,33333333	0,00652174	0,96735847
CL00068	SNORD62	0,33333333	0,00652174	0,96735847
CL00071	SNORD88	0,33333333	0,00652174	0,96735847
CL00002	RNaseP	0,33333333	0,00304569	0,39855097
CL00083	mir-2	0,33333333	0,00200267	0,94367721
CL00028	SNORA13	0,33333333	0,00173611	0,90999479
CL00042	SNORA65	0,33333333	0,00164564	0,90576014
CL00062	SNORD49	0,33333333	0,00164564	0,90576014
CL00031	SNORA21	0,33333333	0,00160514	0,86744392
CL00005	U1	0,33333333	0,00152827	0,66457625
CL00004	Telomerase	0,33333333	0,00152284	0,39855097
CL00014	CRISPR-1	0,3	0,07	0,9871821
CL00096	mir-290	0,28571429	0,00487805	0,9617419
CL00045	SNORD15	0,25	0,04878049	0,97382537
CL00034	SNORA35	0,25	0,03225806	0,95853922
CL00063	SNORD52	0,25	0,01197605	0,97472972
CL00093	mir-182	0,25	0,01007557	0,97282105
CL00053	SNORD31	0,25	0,00869565	0,96735847
CL00027	SNORA9	0,25	0,00231481	0,90999479
CL00035	SNORA36	0,25	0,00231481	0,90999479
CL00032	SNORA27	0,25	0,00214018	0,86744392
CL00040	SNORA62	0,25	0,00214018	0,86744392
CL00038	SNORA52	0,22222222	0,00479904	0,93967039
CL00066	SNORD60	0,2	0,01086957	0,96735847
CL00057	SNORD39	0,16666667	0,03529412	0,98681884
CL00051	SNORD29	0,16666667	0,02391304	0,96735847
CL00069	SNORD74	0,16666667	0,00600801	0,94367721
CL00021	SNORA2	0,16666667	0,00347222	0,90999479
CL00001	tRNA	0,16666667	0,00304569	0,39855097
CL00102	group-II-D1D4	0,14285714	0,00363636	0,83190149
CL00054	SNORD33	0,125	0,0173913	0,96735847
