# Supplementary material for: Clustering Rfam 10.1: Clans, Families, and Classes
Source: Genes (Basel). 2012 Jul 5;3(3):378–90. doi: 10.3390/genes3030378 (PMC3899987; doi:10.3390/genes3030378)
Supplement: Supplementary File 3 — PDF-Document (PDF, 44 KB) [file genes-03-00378-s003.pdf]

ClanAC	ClanN	Clan members
CL00003	7	[RF00017,RF00169,RF01854,RF01855,RF01857,RF01502,RF01856]
CL00015	4	[RF01318,RF01320,RF01376,RF01377]
CL00019	2	[RF00426,RF00423]
CL00048	2	[RF00569,RF01183]
CL00024	2	[RF00392,RF01240]
CL00089	2	[RF00672,RF00824]
CL00099	2	[RF00643,RF00692]
CL00090	2	[RF00830,RF00831]
CL00097	2	[RF00866,RF00363]
CL00007	2	[RF00015,RF00618]
CL00092	2	[RF00694,RF00859]
CL00006	2	[RF00004,RF00007]
CL00073	2	[RF00609,RF00046]
CL00052	2	[RF00088,RF01283]
CL00029	2	[RF00560,RF00416]
CL00084	2	[RF00716,RF00818]
CL00025	2	[RF00409,RF01246]
CL00047	2	[RF00093,RF01159]
CL00055	2	[RF00147,RF01205]
CL00072	2	[RF00055,RF01299]
CL00076	2	[RF00610,RF01280]
CL00008	2	[RF01277,RF00206]
CL00050	2	[RF00136,RF00087]
CL00060	2	[RF00287,RF00359]
CL00064	2	[RF00151,RF00608]
CL00074	2	[RF00186,RF00339]
CL00075	2	[RF00584,RF01173]
CL00077	2	[RF00289,RF01199]
CL00088	2	[RF00685,RF00794]
CL00091	2	[RF00727,RF00728]
CL00087	2	[RF00711,RF00456]
CL00094	2	[RF00654,RF00747]
CL00095	2	[RF00754,RF00948]
CL00010	2	[RF00163,RF00008]
CL00049	2	[RF01188,RF00054]

CL00079	2	[RF01287,RF01235]
CL00081	2	[RF01210,RF00304]
CL00020	2	[RF00198,RF00199]
CL00085	2	[RF00254,RF00455]
CL00016	2	[RF00243,RF00107]
CL00100	4	[RF00012,RF01846,RF01847,RF01848]
CL00046	2	[RF00138,RF01216]
CL00070	2	[RF00309,RF00591]
CL00080	2	[RF01279,RF00338]
CL00086	2	[RF00655,RF00917]
CL00018	2	[RF00422,RF00565]
CL00030	2	[RF00429,RF00401]
CL00011Glm	2	[RF00083,RF00128]
CL00026	2	[RF00393,RF01257]
CL00009	2	[RF00026,RF00619]
CL00012	2	[RF00162,RF00634]
CL00039	2	[RF00417,RF01248]
CL00044	2	[RF00581,RF01249]
CL00059	2	[RF00221,RF01238]
CL00043	2	[RF00090,RF01263]
CL00022	2	[RF00334,RF01260]
CL00023	2	[RF00394,RF01264]
CL00017	2	[RF00061,RF00209]
CL00061	2	[RF00218,RF01259]
CL00082	2	[RF01296,RF01294]
CL00098	2	[RF00645,RF00865]
CL00101	2	[RF00174,RF01482]
CL00041	2	[RF00264,RF01267]
CL00078	2	[RF00045,RF01271]
CL00013	2	[RF00100,RF01052]
CL00037	2	[RF00554,RF01272]
CL00036	4	[RF00405,RF00418,RF01237,RF01245]
CL00033	3	[RF00400,RF00545,RF01269]
CL00067	3	[RF00270,RF01170,RF01200]
CL00056	3	[RF00211,RF01207,RF00328]
CL00058	3	[RF00274,RF00588,RF01214]

CL00065	3	[RF00273,RF00473,RF00160]
CL00068	3	[RF00153,RF00205,RF01218]
CL00071	3	[RF00604,RF01424,RF01209]
CL00002	6	[RF00010,RF00009,RF00011,RF00030,RF00373,RF01577]
CL00083	3	[RF00047,RF00143,RF00813]
CL00028	3	[RF00396,RF01255,RF01438]
CL00042	3	[RF00302,RF01292,RF01254]
CL00062	3	[RF00277,RF00337,RF01300]
CL00031	3	[RF00412,RF01258,RF01437]
CL00005	3	[RF00003,RF00548,RF00488]
CL00004	3	[RF00024,RF01050,RF00025]
CL00014	7	[RF01315,RF01317,RF01327,RF01338,RF01352,RF01379,RF01328]
CL00096	4	[RF01413,RF00639,RF00665,RF00668]
CL00045	4	[RF00067,RF01185,RF01226,RF01223]
CL00034	4	[RF00598,RF00407,RF00430,RF00566]
CL00063	4	[RF00325,RF00276,RF00333,RF01176]
CL00093	4	[RF00663,RF00706,RF00702,RF00843]
CL00053	4	[RF00089,RF00266,RF01281,RF01177]
CL00027	4	[RF00411,RF01256,RF01243,RF01436]
CL00035	4	[RF00340,RF01242,RF01262,RF01439]
CL00032	4	[RF00568,RF00443,RF01265,RF01440]
CL00040	4	[RF00091,RF01261,RF01251,RF01434]
CL00038	8	[RF00425,RF00155,RF00419,RF01224,RF01239,RF01252,RF00307,RF01435]
CL00066	5	[RF00271,RF00345,RF00527,RF00471,RF01194]
CL00057	6	[RF00571,RF00157,RF01178,RF00358,RF01181,RF00268]
CL00051	11	[RF00049,RF00476,RF01203,RF00212,RF00070,RF00592,RF00135,RF01302,RF00479,RF01198,RF00475]
CL00069	9	[RF00181,RF01169,RF00284,RF00530,RF00152,RF00357,RF01278,RF00570,RF00509]
CL00021	6	[RF00410,RF00190,RF00544,RF01253,RF01250,RF01441]
CL00001	6	[RF00005,RF00023,RF01849,RF01852,RF01851,RF01850]
CL00102	7	[RF02001,RF01998,RF02012,RF02005,RF02004,RF01999,RF02003]
CL00054	8	[RF00133,RF00134,RF00532,RF00535,RF00280,RF00472,RF01201,RF01197]
